# Selenium Intake and Postnatal Depression—A Short Review

**DOI:** 10.3390/nu16121926

**Published:** 2024-06-18

**Authors:** Natalia Karkoszka, Ewa Gibula-Tarlowska, Jolanta Kotlinska, Anna Bielenica, Kinga Gawel, Ewa Kedzierska

**Affiliations:** 1 Department of Pharmacology and Pharmacodynamics, Medical University of Lublin, 4a Chodźki Street, 20-400 Lublin, Poland; ewa.gibula-tarlowska@umlub.pl (E.G.-T.); jolanta.kotlinska@umlub.pl (J.K.); ewa.kedzierska@umlub.pl (E.K.); 2 Department of Biochemistry, Medical University of Warsaw, 1 Banacha Street, 02-097 Warsaw, Poland; anna.bielenica@wum.edu.pl; 3 Department of Experimental and Clinical Pharmacology, Medical University of Lublin, 8b Jaczewskiego Street, 20-090 Lublin, Poland; kingagawel@umlub.pl

**Keywords:** selenium, postnatal depression, postpartum depression

## Abstract

Postnatal depression is a common and severe complication of childbirth. It is an important public health problem with significant implications for both mothers and children. The exact mechanisms underlying and the factors influencing the occurrence of postnatal depression remain unclear. The literature suggests that certain dietary deficiencies during pregnancy and the postnatal period may contribute to a greater risk of maternal depression. This review focuses on the role of selenium in postnatal depression. It collects evidence from published interventional and observational studies investigating the relationship between selenium intake during the antenatal and postnatal periods and the mental status of postpartum women and summarises information about biological mechanisms that may underlie the association between selenium status and postnatal depression. The review includes studies identified through electronic searches of Medline (via PubMed) and Google Scholar databases until December 2023. Despite the small number of relevant studies and their potential methodological limitations, the findings suggest that optimizing selenium status may support the prevention and treatment of postnatal depression. Further longitudinal and interventional studies are necessary to confirm the clinical significance of these effects.

## 1. Introduction

Postnatal depression is a depressive disease that affects 10–20% of women after childbirth, with significant variability in prevalence between countries and geographic regions [1,2]. In a recent meta-analysis by Wang et al. that comprehensively synthesized data on the global prevalence of postnatal depression, Southern Africa was identified as the region with the highest rate (39.96%), followed by Western Asia (19.83%). In the same meta-analysis, among countries with at least five relevant studies, South Africa had the highest prevalence of postnatal depression in women (38.79%), while Spain had the lowest rate (9.09%) [2].

The latest editions of disease classification systems by the American Psychiatric Association and the World Health Organization, i.e., the Diagnostic and Statistical Manual of Mental Disorders (DSM-5) and the International Statistical Classification of Diseases (ICD-11) define postnatal depression as mental or behavioural disorders associated with pregnancy, childbirth, or the puerperium (about six weeks after childbirth) [3,4]. However, other clinicians and specialists say that a mother can suffer from postnatal depression even within the first 12 months after giving birth [5,6].

Postnatal depression can severely impair a woman’s ability to function in daily life. Unlike the “baby blues”, which typically occur a few days postpartum and resolve within a few weeks, postnatal depression most commonly begins within the first three months after delivery. It lasts longer and often requires medical treatment [7]. The symptoms include depressed mood or severe mood changes, sleep and appetite disturbances, anxiety, fatigue, feelings of guilt, and preoccupation. The symptoms are more severe than those of the “baby blues” and can significantly affect the relationship between the mother and child [8,9]. In the most severe cases, postnatal depression may lead to suicidal thoughts. In a recent retrospective cohort study of more than 22,000 women screened for depression during the perinatal period, 3.8% reported suicidal ideation [10]. Pathophysiological mechanisms underlying postnatal depression are diverse. The roles of neuroendocrine alterations, disruptions in neurotransmission, circuit dysfunction, neuroinflammatory processes, and oxidative stress over the course of gestation, as well as genetic and epigenetic factors, have been discussed [11,12].

**Figure 1:**
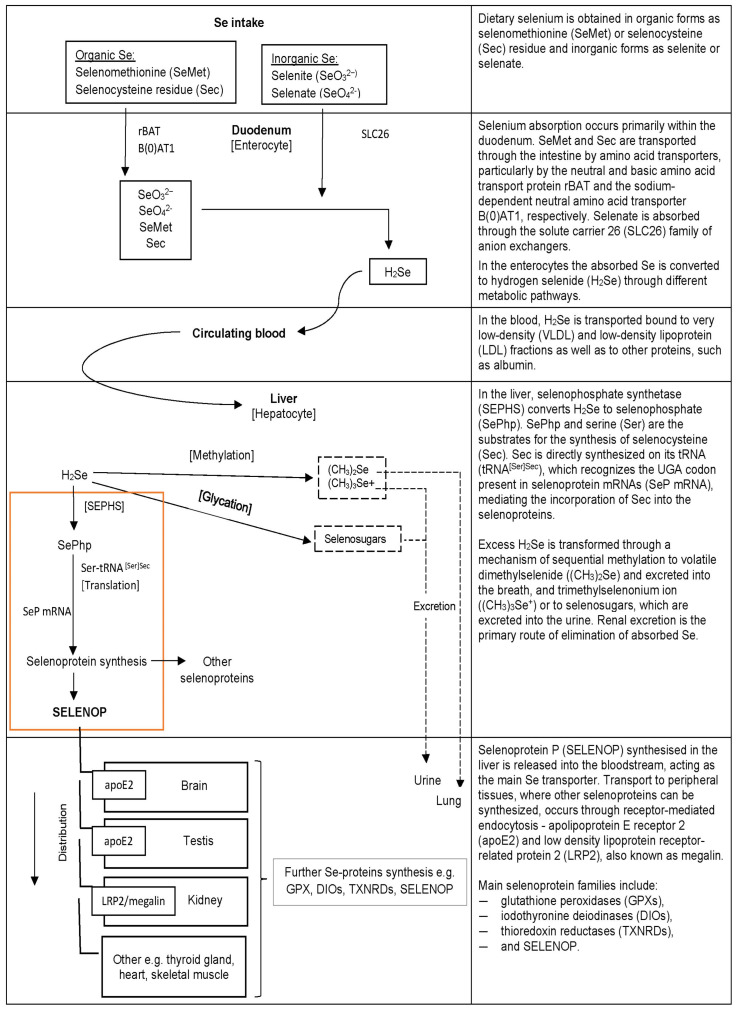
Selenium metabolism and selenoprotein synthesis in the human body [15,18,19,20,21].

Selenium is an essential trace element with antioxidant properties relevant to several metabolic pathways. Dietary selenium from plant and animal sources mainly occurs in organic forms, primarily selenomethionine and selenocysteine. In water, selenium is predominantly present in inorganic form as selenate [13]. Selenium from organic or inorganic sources is absorbed in the gastrointestinal tract and subsequently transported to the liver, where it is metabolised and used for the synthesis of selenoproteins (Figure 1). Selenium, as a selenocysteine, is incorporated at the active site of a wide range of selenoproteins, such as glutathione peroxidases, thyroid hormone deiodinases, thioredoxin reductases, and selenoprotein P [14]. The biological functions of human selenoproteins are diverse, and they play key roles in cellular antioxidant defence and maintenance of redox homeostasis [15]. The majority of selenium in the human body is stored in skeletal muscles, whereas the highest concentration is maintained by the thyroid gland [16]. Furthermore, the importance of selenium in brain function was indicated in animal studies, which revealed the preferential retention of selenium in the neuronal tissues of rodents on a selenium-deficient diet [17].

Adequate selenium intake is essential for human health. As a constituent of selenoproteins, selenium plays important roles in DNA synthesis and repair [22], the modulation of cell proliferation [23,24], the protection from oxidative stress, reproduction, thyroid hormone metabolism, and the optimal functioning of the immune system [25]. Selenium deficiency has been implicated in cardiac and skeletal muscle disorders [26,27], neurological disorders [28,29], altered immune responses to viral infections [29,30], impaired antitumour immunity [31], and infertility in both males and females [32,33,34]. In pregnant women, inadequate selenium status is associated with gestational complications, miscarriages, preterm birth [33,35], and an increased risk of preeclampsia, pregnancy-induced hypertension [36], and gestational diabetes [37,38]. Additionally, animal and human studies have indicated the effects of selenium on modulating cognitive performance [39,40] and mood [41], as well as its role in depression [42].

The US Recommended Dietary Allowance (RDA) of selenium for adults, both women and men, is 55 µg/day [43]. In the UK, selenium RDA is defined as 75 µg/day for men and 60 µg/day for women [44]. The optimum range of serum/plasma selenium levels is estimated to be between 70 μg/L and 90 μg/L [43,45]. The recommended doses are based on the amount of selenium required to maximise the synthesis of glutathione peroxidase [43]. However, recent studies have indicated that selenoprotein P is a more appropriate biomarker for assessing the optimal expression of all selenoproteins. Both glutathione peroxidase and selenoprotein P plasma concentrations reach a plateau when the selenium supply is sufficiently high; however, a higher intake (approximately 100–150 µg/day) is required to optimise the expression of selenoprotein P [46]. Selenium requirements are higher during pregnancy and lactation because of both the developing foetus and maternal physiological and hormonal changes [47], and several studies have reported a decrease in serum selenium during gestation [48,49,50]. It has been estimated that pregnant and lactating women need about 60 and 70 µg/day, respectively [43].

Based on the scientific opinion of the Panel on Nutrition, Novel Foods and Food Allergens of the European Food Safety Authority, the upper limit for selenium is 255 μg/day for all adults, including pregnant or lactating women, 45 μg/day for infants 4–6 months, and 55 μg/day for infants 7–11 months. Upper intake levels for children from 1 to 17 years range from 70 to 230 μg/day, depending on age [13].

While selenium deficiency during pregnancy has been linked to adverse outcomes such as poor foetal growth [51], low birth weight [52], impaired immune function [53], and declines in children’s psychomotor, language, and cognitive development [54,55,56], an excess of selenium during the prenatal period is equally undesirable and may contribute to negative outcomes. A recent prospective birth cohort study indicated a possible association between prenatal exposure to high maternal selenium levels and the child’s risk of developing autism spectrum disorder and attention deficit hyperactivity disorder (ADHD) [57]. It should be noted that the evidence on the association between maternal selenium status during pregnancy and adverse outcomes for foetal or further child development is conflicting, and there is not enough evidence to conclude a causal relationship. Considering the potential adverse effects of both low and high selenium levels during pregnancy and the postnatal period for both mother and infant, it is essential to maintain optimal selenium levels in maternal nutrition, avoiding deficiencies or excessive intake by accounting for dietary selenium sources.

Brazil nuts are among the richest sources of selenium, with exceptionally high concentrations (approximately 1920 μg/100 g; 96 μg per 1 nut). One Brazil nut can provide more than the recommended daily intake of selenium for adults; therefore, pregnant women should consume them in moderation. Other selenium-rich foods include fish and shellfish (e.g., tuna, 75–108 μg/100 g; sardines 34–52 μg/100 g; halibut 36–55 μg/100 g; shrimps 19–45 μg/100 g; salmon 22–46 μg/100 g), offal, meat and poultry (e.g., beef liver, 28–50 μg/100 g; turkey, 15–30 μg/100 g; chicken breast 18–24 μg/100 g), eggs (16–30 μg/100 g), whole grains (e.g., brown rice, 8–17 μg/100 g), whole wheat bread (26–30 μg/100 g), seeds and nuts (e.g., sunflower seeds, 51–58 μg/100 g), certain mushrooms (e.g., shiitake mushrooms, 18–25μg/100 g), followed by milk and dairy products, which contain smaller amounts (e.g., milk 1.7–3.7 μg/100 g) [58,59]. These values are approximate and can vary based on specific food sources as well as preparation methods. The selenium content in animal-derived products, including dairy and eggs, depends on the selenium levels in the animals’ feed, while in plants, it is determined by the selenium concentration in the soil where they are grown. The USDA’s Food Data Central and the European Food Safety Authority’s food composition database provide comprehensive information on foods containing selenium [58,59]. Nevertheless, due to significant regional variability in selenium concentrations in foods, it is important to consider regional or country-specific reference sources. Importantly, while this review focuses on the role of selenium, broader consideration should be given to overall maternal nutritional status since other dietary deficiencies during pregnancy and the postnatal period may also contribute to a greater risk of maternal depression.

## 2. Methods

This review aimed to identify and summarize the available evidence on selenium intake during the antenatal and postnatal periods, its impact on maternal mental health, and the risk of developing postnatal depression. A non-systematic literature review was conducted using electronic searches of the Medline (via PubMed) and Google Scholar databases to identify relevant publications. The initial search targeted interventional or observational studies investigating the relationship between selenium intake and postpartum women’s mental status, using keywords such as ‘selenium’, ‘nutrients’, ‘trace elements’, ‘micronutrients’, along with ‘postnatal depression’, ‘postpartum depression’, ‘postpartum depressive symptoms’, and ‘perinatal depression’, until December 2023. Studies reporting nutrient supplementation, nutritional status, or dietary intakes during pregnancy in the context of postnatal depression were further reviewed to identify if selenium-specific outcomes were reported and, if so, were included in this review. Additionally, focused searches were performed to explore the possible underlying mechanisms and biological plausibility of the beneficial effects of selenium on postnatal depression.

## 3. Selenium Intake and Its Effects on Maternal Mental Health Outcomes Postpartum

Interventional clinical studies reporting the effects of selenium supplementation during the perinatal period on postpartum maternal mental health outcomes are currently limited to a single randomised, placebo-controlled study. The remaining relevant published studies that evaluated the association between selenium intake and postnatal depression included one case–control study and two prospective cohort studies. An overview of the studies and reported selenium-related outcomes is provided in Table 1.

The studies used the Edinburgh Postnatal Depression Scale (EPDS), a 10-item questionnaire developed to identify women suffering from postnatal depression. Items of the scale (each scored on a scale of 0 to 3) correspond to various clinical symptoms of depression, such as anhedonia, feelings of guilt, sleep disturbances, low energy, and suicidal thoughts. The overall assessment is calculated by summing up the scores for each of the ten items. Higher scores indicate more depressive symptoms. Mothers scoring above 12 are likely to be suffering from depression and should seek medical advice. EPDS ≥ 10 suggests a possible presence of depression [60].

**Table 1:** Overview of studies reporting the effects of selenium on maternal mental health outcomes during the postnatal period.

Study Design	Country	Sample Size	Study Population	Se Dose/Intake	Depression Assessment Method	Selenium-Related Outcome	Ref.
Randomised, double-blind, placebo-controlled study	Iran	*n* = 85 Selenium group *n* = 44 Placebo group *n* = 41	Women aged 16–35 years, primigravid, with gestational age up to 12 weeks. Mean age (±SD)— Selenium group: 21.64 (±2.45) Placebo group: 21.59 (±3.40)	100 μg/day (administered as selenium yeast tablets from the first trimester until birth)	EPDS Collected within 8 weeks of delivery.	Selenium supplementation was associated with a significant increase in mean serum selenium concentration at term (*p* < 0.001). Serum selenium concentration remained statistically unchanged by the end of the trial in the control group (*p* > 0.05). The mean EPDS score in the selenium group was significantly lower than that in the control (placebo) group—8.8 ± 5.1 vs. 10.7 ± 4.4, respectively (*p* < 0.05).	[61]
Cohort study	Canada	*n* = 475	Pregnant women aged 16 years and older, with gestational age ≤27 weeks. Mean age (95% CI) — Women with postpartum EPDS score < 10 (*n* = 416): 31.2 (30.8–31.6) Women with postpartum EPDS score ≥ 10 (*n* = 59): 31.6 (30.3–32.8)	Mean maternal selenium intake from supplementation (±SD) — Women with postpartum EPDS score < 10 (*n* = 416): 25 (±17) μg/day Women with postpartum EPDS score ≥ 10 (*n* = 59): 19 (±13) μg/day	EPDS Collected at each trimester and 12 weeks postpartum.	The mean supplementary intake of selenium was significantly higher in women with EPDS < 10 than in those with EPDS ≥ 10; (*p* = 0.0015). Selenium intake from supplements was negatively associated with postpartum depressive symptoms.	[62]
Case–control study	Iran	*n* = 163 Case group: *n* = 81 (women diagnosed with postpartum depression, PPD) Control group: *n* = 82 (non-PPD)	Women aged 18 to 45 years in postpartum period. Mean age (±SD)— Case group: 28.4 (± 6.7) Control group: 27.2 (± 5.6)	Median dietary maternal selenium intake assessed using semi-quantitative food frequency questionnaire; adjusted for total energy intake. Case group: 6.5 μg/day Control group: 23.7 μg/day	EPDS Case group included women with EPDS scores > 12 during period from 1 month to 6 months postpartum.	Dietary intake of selenium was significantly lower in the PPD group than in the non-PPD control (*p* < 0.001). Higher intake of selenium was associated with reduced odds of post-partum depression.	[63]
Cohort study	New Zealand	*n* = 87	Breastfeeding women aged 16 years and older at 3 months postpartum. Mean age (±SD)— 31.5 ± 4.2	Median maternal selenium intake estimated from weighed four-day diet diary (including supplements)—62 μg/day. Note: 5 out of 87 women were supplemented with selenium, ranging from 25 to 65 μg/day.	EPDS Collected at 3, 6, and 12 months postpartum.	No significant association between plasma selenium values and EPDS scores was observed.	[64]

The randomised, placebo-controlled study conducted by Mokhber et al. investigated the effect of selenium supplementation (100 μg/day) taken from the first trimester until childbirth in preventing postnatal depression. The EPDS was completed by the study participants within eight weeks of delivery. The study reported significantly lower EPDS scores in a group of pregnant women supplemented with selenium compared to those in the control group taking a placebo [61]. Adjustment for confounding variables, including dietary selenium intake, was not reported. Similarly, Leung et al. examined the relationship between micronutrient supplementation and the risk of occurrence of symptoms of postpartum depression in women in the Alberta Pregnancy Outcomes and Nutrition (APrON) study [62]. The mean nutrient intake from supplements was generally higher in women with lower EPDS scores. Additionally, the mean intake of selenium was found to be significantly different between women with EPDS <10 and women with EPDS ≥10 (*p* = 0.0015), where an EPDS ≥ 10 is considered indicative of the possible presence of depression. It is worth noting that dietary selenium intake was not included in the analysis, and serum levels of selenium were not available [62]. A case–control study by Amini et al. investigated the association between dietary nutrient intake during pregnancy and the incidence of postpartum depression (PPD). In this study, higher selenium intake was associated with reduced odds of postpartum depression after adjusting for body mass index (BMI) and total energy intake (*p* < 0.001) [63].

A recently published meta-analysis by Sajjadi et al. synthesised the results of the aforementioned studies by Amini et al. and Leung et al. and showed a significant inverse association between selenium intake and the risk of postpartum depression [42]. Interestingly, Sajjadi et al. demonstrated no significant association between selenium intake and the risk of other types of depression [42]. In contrast, the results of the Mother and Infant Nutrition Investigation (MINI), an observational longitudinal cohort study, remain inconclusive regarding the correlation between selenium status and the risk of postnatal depression and anxiety. The study investigated maternal and infant selenium intake and status during the first year postpartum. No significant association between plasma selenium levels and EPDS scores was reported; however, the authors noted that the majority of study participants might have had sufficient selenium levels to meet the saturation of glutathione peroxidase activity. The study, however, did not measure functional selenium status [64].

Despite their heterogeneity, the results of these studies suggest that selenium supplementation may prevent and support the treatment of postnatal depression. Importantly, considering the limited geographical coverage of the studies (including Iran, Canada, and New Zealand), it is difficult to assess whether the results may be generalised, given that selenium status in humans was found to be highly influenced by environmental selenium status and may vary significantly between countries or regions [65]. Additionally, limitations noted by the respective researchers depending on the type of study included bias of the selected study population/cohort, missing information on serum/plasma selenium or lack of measurement of functional selenium status, risk of non-compliance during the supplementation, and limitations of food questionnaires including recall bias and potential measurement errors [61,62,63,64]. Thus, further studies are required to confirm the clinical significance of these observed effects.

## 4. Biological Mechanisms That Might Underlie Selenium’s Effects in Postanal Depression

Several biological mechanisms have been hypothesised to explain the beneficial effects of selenium on depression. The modulatory effects of selenium on various brain signalling pathways, including the serotonergic, dopaminergic, and noradrenergic systems [66,67,68], as well as its protective effects against glutamate toxicity [69], have been described. Furthermore, the indirect interaction of selenium with redox signalling mechanisms [70,71], inflammatory modulation, and participation in neuronal metabolism may provide a biologically accurate explanation for the effects of selenium [68].

Selenium plays a pivotal role in the regulation of inflammatory and oxidative responses. As a cofactor in key enzymes involved in cellular antioxidant activity, it plays an important role in the defence against oxidative stress [72]. During pregnancy, the increased oxygen demand enhances the formation of reactive oxygen species (ROS) and products of ROS-induced lipid peroxidation [49,73]. Furthermore, plasma selenium concentration and glutathione peroxidase activity decrease during this period [74]. Inadequate selenium status may result in an imbalance between the production of ROS and their neutralisation by protective mechanisms. Optimum intake is critical because both selenium deficiency and excess can induce oxidative stress and affect cellular redox homeostasis, leading to inflammation [75]. This mechanism potentially links the effects of selenium to the inflammatory hypothesis of depression. High expression of proinflammatory cytokines (such as interleukin (IL)-1β, TNF-α, and IL-6) has been observed in inflammation models of depression in rodents. Furthermore, the results of a recent prospective cohort study on the relationship between oxidative stress and postpartum depression showed a significant inverse correlation between serum total antioxidant capacity and EPDS scores [12]. A recent meta-analysis of studies that reported immune marker levels in the peripheral blood of patients with depression and matched the healthy controls revealed significant elevations in the levels of several proinflammatory cytokines and C-reactive protein in patients with depression [76]. Proinflammatory cytokines activate the kynurenine pathway of tryptophan catabolism by inducing indoleamine 2,3-dioxygenase (IDO) (the first and rate-limiting enzyme of the kynurenine pathway). When the kynurenine pathway is stimulated, less tryptophan is available for serotonin synthesis [77]. Furthermore, neuroactive metabolites such as quinolinic acid (QUIN) are generated and can significantly influence the regulation of dopamine and glutamate neurotransmission [78]. Maes et al. observed an increased kynurenine/tryptophan ratio (indicating IDO activity) at the end of pregnancy, with further increases in early puerperium. Moreover, increases in plasma kynurenine and kynurenine/tryptophan ratio were found to be more pronounced in women whose anxiety and depression scores significantly increased during puerperium [79].

Blocking the synthesis of certain inflammatory cytokines has been investigated as a viable option for patients with depression accompanied by increased inflammation. Given that IL-6 levels were demonstrated to be inversely correlated with the serum concentrations of selenium and supplementation with selenium was shown to reduce serum CRP, it is plausible that the improvement of depressive symptoms following selenium supplementation may be linked to its anti-inflammatory properties [80,81,82]. In postnatal depression, an anti-inflammatory effect might also be of particular relevance, given that notable changes in serum proinflammatory mediators are observed during pregnancy and the postpartum period [82]. The concentration of TNF-α was found to be elevated in peripheral blood during the entire gestation period [83,84,85,86], whereas IL-6 and IL-8 levels were observed to increase from the second to the third trimester [86,87]. Christian et al. reported increases in the levels of IL-6, TNF-α, IL-8, and IL-1β at 4–6 weeks postpartum compared to those in the third trimester of pregnancy [88]. In another study, women with anxiety and depressive symptoms in puerperium were characterised by increased activation of the inflammatory response system and reduced anti-inflammatory activity in the serum [89].

Selenium can also alter the symptoms of depression by affecting thyroid function, as it is essential for the adequate metabolism of thyroid hormones. Selenium is incorporated into iodothyronine deiodinases, which catalyse the conversion of thyroxine (T4) to metabolically active triiodothyronine (T3) [90]. Additionally, glutathione peroxidase is essential for protecting the thyrocytes from excess H_2_O_2_ [91]. Selenium deficiency and the consequent thyroid malfunction may play a role in the development of depression. A recent systematic review of the relationship between maternal thyroid changes and postpartum depression remains inconclusive regarding the mechanisms of thyroid function that might be involved in the pregnancy–puerperal cycle and postpartum depression [92]. However, the authors indicated that a positive thyroperoxidase antibody status may be a possible biomarker of vulnerability to depression during pregnancy and the postpartum period [92,93]. A systematic review and meta-analysis conducted by Fan et al. provided evidence of an association between selenium supplementation and a decrease in thyroid peroxidase autoantibody (TPOAb) and thyroglobulin antibody (TgAb) levels in patients with autoimmune thyroiditis, with a significant reduction in titres after 6 and 12 months of supplementation, respectively; additionally, the authors indicated that patients after selenium supplementation had a higher chance of improving their mood or well-being compared with controls [94]. While selenium is one of the micronutrients required for thyroid hormone metabolism, other essential trace elements, including iodine, iron and zinc, play an important role in the synthesis and metabolism of thyroid hormones. Therefore, broader consideration should be given to overall micronutrient status to ensure optimal thyroid function [95,96].

Another potential mechanism that may mediate the protective role of selenium against depression involves its effect on brain-derived neurotrophic factor (BDNF). BDNF regulates neuronal network plasticity, which is disrupted during depression [97,98]. According to the neurotrophic hypothesis of depression, decreased levels of BDNF and other trophic factors may affect neurogenesis and impair synaptic transmission in the hippocampus and prefrontal cortex, thus mediating mood and emotions [99,100]. Although the effect of selenium on BDNF expression is not well characterised in humans, decreased cerebral BDNF mRNA levels have been observed in selenium deficiency in rats [101]. In contrast, in a more recent study, no significant change in BDNF levels was identified in mouse serum after administration of sodium selenite; however, the authors noted that the short duration of the stress factor might have contributed to the lack of the observed effect [102]. In another recent study, researchers demonstrated the protective effects of sodium selenite on brain injury induced by mercuric chloride through inhibition of apoptosis and inflammation via the activation of the brain-derived neurotrophic factor (BDNF)/tropomyosin-related kinase receptor type B (TrKB)/phosphatidylinositol 3-kinase (PI3K)/protein kinase B (AKT) signalling pathway and the suppression of the nuclear factor kappa B (NF-κB) signalling pathway in chicken brain tissues [103]. Notably, the role of impaired BDNF signalling via the TrKB receptor in mood disorders, as well as the effects of antidepressants on BDNF synthesis and signalling, have been broadly described in the literature [104,105,106,107,108,109,110]. Importantly, in the context of postnatal depression, Lee et al. identified significant changes in BDNF plasma concentrations in women with depression during the perinatal period. The plasma concentration of BDNF observed in this study was significantly higher in healthy pregnant women than in healthy non-pregnant controls and significantly lower in women with postpartum depression than in women in the perinatal non-depressed control group. In addition, in the postpartum depression-recovery group, a significant increase in BDNF levels was observed at 6 weeks after delivery compared with the levels observed in this group at 24 weeks of gestation [111]. Gao et al. demonstrated a relationship between reduced serum BDNF levels and the risk of postpartum depression in a 3-month follow-up study [112].

Finally, the impact of selenium intake on the gut microbiota and its downstream effects on the gut–brain axis cannot be ruled out [18]. Differences in the gut microbial composition of patients with postpartum depressive disorder compared with that of healthy controls were demonstrated by Zhou et al. [113].

**Figure 2:**
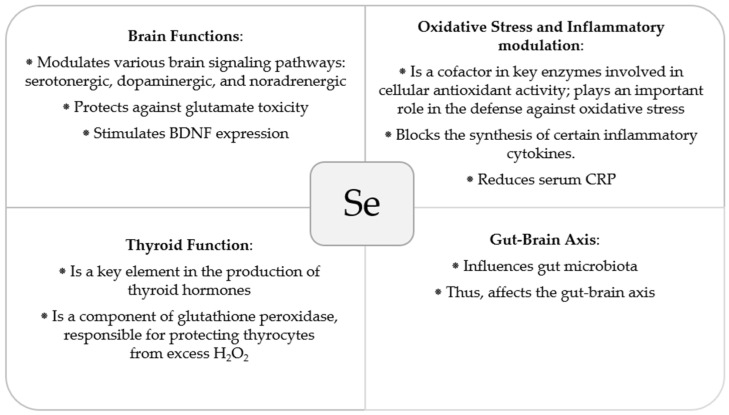
Overview of biological mechanisms that may underlie the association between selenium status and postnatal depression [18,66,67,68,69,72,76,90,91].

In summary, selenium may exert its effects on several aspects that may be relevant to postnatal depression (Figure 2).

## 5. Limitations

This review outlines the available evidence on selenium intake during the antenatal and postnatal periods and its impact on the risk of developing postnatal depression. However, there are several limitations. Firstly, there are only a small number of studies. Specifically, there is only one randomized, placebo-controlled study on the effects of selenium supplementation in preventing postnatal depression. Additionally, only a few other studies reporting selenium-related outcomes in the context of postnatal depression were identified, including one case–control study and two prospective cohort studies. The results of these studies are heterogeneous, complicating the analysis of findings and conclusions. Moreover, while this review focuses specifically on selenium, nutrients consumed together can influence each other through interactive or synergistic effects, making it challenging to assess the impact of selenium alone. Therefore, more longitudinal and interventional studies are required to confirm the clinical significance of the observed effects of selenium on postnatal depression.

## 6. Conclusions

Studies on the effects of selenium on postnatal depression published in the literature are limited; however, the combined outcomes indicate a protective role of selenium against postnatal depression, particularly in women with nutritional selenium deficiency. The association between selenium deficiency and the risk of postnatal depression appears to be biologically plausible, given the potential relevance of selenium in this state. Further studies are needed to validate these findings across diverse populations. Future research should include standardized selenium supplementation protocols and assess functional selenium status to ensure the comparability of results and provide more evidence regarding the potential role of selenium in the prevention and management of postnatal depression.
